# Ni_2_Sr(PO_4_)_2_·2H_2_O

**DOI:** 10.1107/S1600536810045113

**Published:** 2010-11-27

**Authors:** Abderrazzak Assani, Mohamed Saadi, Mohammed Zriouil, Lahcen El Ammari

**Affiliations:** a Laboratoire de Chimie du Solide Appliquée, Faculté des Sciences, Université Mohammed V-Agdal, Avenue Ibn Battouta, BP 1014, Rabat, Morocco

## Abstract

The title compound, dinickel(II) strontium bis­[ortho­phosphate(V)] dihydrate, was obtained under hydro­thermal conditions. The crystal structure consists of linear chains _∞_^1^[NiO_2/2_(OH_2_)_2/2_O_2/1_] of edge-sharing NiO_6_ octa­hedra (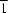 symmetry) running parallel to [010]. Adjacent chains are linked to each other through PO_4_ tetra­hedra (*m* symmetry) and arranged in such a way to build layers parallel to (001). The three-dimensional framework is accomplished by stacking of adjacent layers that are held together by SrO_8_ polyhedra (2/*m* symmetry). Two types of O—H⋯O hydrogen bonds involving the water mol­ecule are present, *viz*. one very strong hydrogen bond perpendicular to the layers and weak trifurcated hydrogen bonds parallel to the layers.

## Related literature

For catalytic properties of phosphates, see: Cheetham *et al.* (1999 ▶); Clearfield (1988 ▶). The crystal structure of anhydrous Ni_2_Sr(PO_4_)_2_ has been reported by El Bali *et al.* (1993 ▶). For crystal structures of some hydrous orthophosphates of divalent cations, see: Assani *et al.* (2010 ▶); Effenberger (1999 ▶); Lee *et al.* (2008 ▶); Britvin *et al.* (2002 ▶); Stock (2002 ▶); Yakubovich *et al.* (2001 ▶). For bond-valence analysis, see: Brown & Altermatt (1985 ▶).

## Experimental

### 

#### Crystal data

Ni_2_Sr(PO_4_)_2_·2H_2_O
*M* *_r_* = 431.01
Monoclinic, 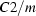

*a* = 8.8877 (3) Å
*b* = 6.0457 (3) Å
*c* = 7.3776 (3) Å
β = 114.173 (2)°
*V* = 361.66 (3) Å^3^
*Z* = 2
Mo *K*α radiation
μ = 12.99 mm^−1^
*T* = 296 K
0.20 × 0.10 × 0.07 mm

#### Data collection

Bruker APEXII diffractometer
Absorption correction: multi-scan (*SADABS*; Bruker, 2005 ▶) *T*_min_ = 0.229, *T*_max_ = 0.403
2376 measured reflections
454 independent reflections
439 reflections with *I* > 2σ(*I*)
*R*_int_ = 0.028

#### Refinement

*R*[*F* ^2^ > 2σ(*F* ^2^)] = 0.019
*wR*(*F* ^2^) = 0.052
*S* = 1.12
454 reflections
49 parameters
3 restraints
H atoms treated by a mixture of independent and constrained refinement
Δρ_max_ = 0.41 e Å^−3^
Δρ_min_ = −0.69 e Å^−3^

### 

Data collection: *APEX2* (Bruker, 2005 ▶); cell refinement: *SAINT* (Bruker, 2005 ▶); data reduction: *SAINT*; program(s) used to solve structure: *SHELXS97* (Sheldrick, 2008 ▶); program(s) used to refine structure: *SHELXL97* (Sheldrick, 2008 ▶); molecular graphics: *ORTEP-3 for Windows* (Farrugia,1997 ▶) and *DIAMOND* (Brandenburg, 2006 ▶); software used to prepare material for publication: *WinGX* (Farrugia, 1999 ▶).

## Supplementary Material

Crystal structure: contains datablocks I, global. DOI: 10.1107/S1600536810045113/wm2418sup1.cif

Structure factors: contains datablocks I. DOI: 10.1107/S1600536810045113/wm2418Isup2.hkl

Additional supplementary materials:  crystallographic information; 3D view; checkCIF report

## Figures and Tables

**Table 1:** Selected bond lengths (Å)

Sr1—O1^i^	2.626 (2)
Sr1—O2	2.636 (3)
Sr1—O3	2.797 (3)
Ni1—O4	2.0285 (19)
Ni1—O1^ii^	2.0499 (19)
Ni1—O3	2.0895 (18)
P1—O2	1.517 (3)
P1—O1	1.539 (2)
P1—O3	1.564 (3)

Symmetry codes: (i) 
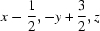
; (ii) 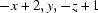.

**Table 2:** Hydrogen-bond geometry (Å, °)

*D*—H⋯*A*	*D*—H	H⋯*A*	*D*⋯*A*	*D*—H⋯*A*
O4—H1⋯O4^iii^	0.85 (6)	2.23 (7)	2.951 (4)	143 (7)
O4—H1⋯O1^iv^	0.85 (6)	2.28 (4)	2.784 (3)	118 (4)
O4—H1⋯O1^v^	0.85 (6)	2.28 (4)	2.784 (3)	118 (4)
O4—H2⋯O2^vi^	0.85 (3)	1.67 (3)	2.511 (4)	169 (9)

Symmetry codes: (iii) 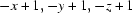; (iv) 
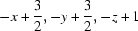
; (v) 
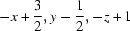
; (vi) 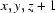.

**Fig. 1.**
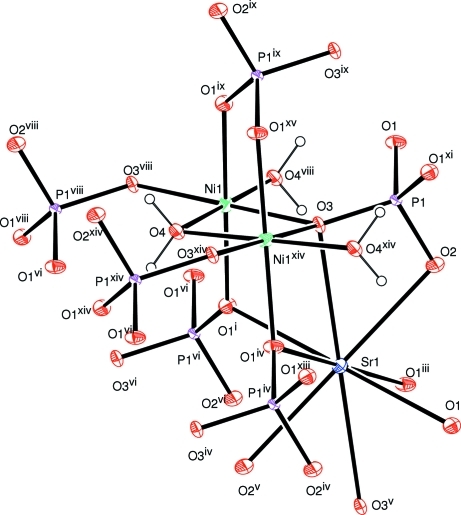
Partial plot of Ni2Sr(PO4)2,2H2O crystal structure. Displacement ellipsoids are drawn at the 50% probability level. Symmetry codes: (i) x - 1/2, -y + 3/2, z; (ii) -x + 3/2, y - 1/2, -z; (iii) -x + 3/2, -y + 3/2, -z; (iv) x - 1/2, y - 1/2, z; (v) -x + 1, -y + 1, -z; (vi) x - 1/2, y + 1/2, z; (vii) -x + 3/2, -y + 1/2, -z; (viii) -x + 3/2, -y + 3/2, -z + 1; (ix) -x + 2, y, -z + 1; (x) x + 1/2, y + 1/2, z + 1; (xi) x, -y + 1, z; (xii) x + 1/2, y - 1/2, z; (xiii) x + 1/2, y + 1/2, z; (xiv) -x + 3/2, y - 1/2, -z + 1; (xv) -x + 2, -y + 1, -z + 1.

**Fig. 2.**
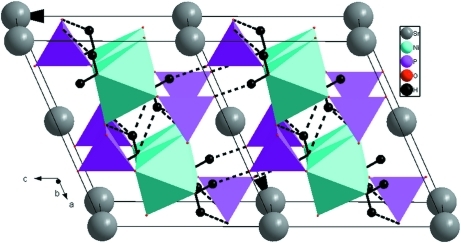
A three-dimensional polyhedral view of the crystal structure of the Ni2Sr(PO4)2.2H2O, showing the stacking of layers along the c axis and the hydrogen bonding scheme (dashed lines).

## supplementary crystallographic information

### Comment

Metal based phosphates continue to be interesting materials due to their remarkable variety of structures, associated with outstanding properties in widespread applications such as catalysis and ion-exchangers (Cheetham *et al.*, 1999; Clearfield, 1988).

Our focus of investigation is associated with mixed divalent orthophosphates with general formula (*M*,*M*')_3_(PO_4_)_2_.*n*H_2_O (Assani *et al.*, 2010). This family of phosphates goes along with a diversity of structures, depending on the size difference of the (various) divalent cations (Effenberger, 1999) and on the degree of hydratation (Yakubovich *et al.*, 2001; Lee *et al.*, 2008), ranging from 0.5 H_2_O in the manganese phosphate Mn_3_(PO_4_)_2_.0.5H_2_O (Stock, 2002) to 22 H_2_O in the case of Mg_3_(PO_4_)_2_.22H_2_O (Britvin *et al.*, 2002). A topological comparison with their corresponding anhydrous phosphates clearly reveals substantial differences in their structural set-up.

By means of the hydrothermal method, we have synthesized a the hydrous strontium nickel phosphate,  Ni_2_Sr(PO_4_)_2_. 2H_2_O, representing a novel structure type. A partial three-dimensional plot of the crystal structure of the title compound is represented in Fig. 1, illustrating the connection of the metal-oxygen polyhedra. The network is built up from three different types of polyhedra more or less distorted, *viz*. SrO_8_ polyhedra (2/*m* symmetry) with distances ranging from 2.636 (2) Å to 2.797 (3) Å, NiO_6_ octahedra (1 symmetry) and PO_4_ tetrahedra (*m* symmetry). Edge-sharing NiO_6_ octahedra form an infinite chain _∞_^1^[NiO_2/2_(OH_2_)_2/2_O_2/1_] running parallel to [010], as shown in Fig. 2. Adjacent chains are connected by PO_4_ tetrahedra and weak trifurcated hydrogen bonds (O4–H1···(O4,O1)) to build up layers parallel to (001). These layers are in turn linked by sheets of Sr^2+^ cations and by very strong hydrogen bond (O4–H2···O2) as shown in Fig. 2 and Table 2.

Bond valence sum calculations (Brown & Altermatt, 1985) for Ni^2+^, Sr^2+^ and P^5+^ ions are as expected, *viz*. 2.03, 1.83 and 4.93 valence units, respectively. The values of the bond valence sums calculated for all oxygen atoms are: 1.83, 1.56 and 1.93 for O1, O2 and O3, respectively. The low bond valence sum of O2 indicates that it is considerably undersaturated and thus acts as an acceptor with a very short H-bond (Table 2).

It is particularly interesting to compare the crystal structures of this compound with that of its corresponding anhydrous phosphate (El Bali *et al.*, 1993). Indeed, both structures can be described by the stacking of two-dimensional layers connected to each other by Sr^2+^ ions. However, we can note the following important differences: The presence of NiO_5_ polyhedra in the anhydrous phase and a different space group (*P*1) and lattice parameters. Moreover, due to the lower symmetry, the polyhedra are more distorted in the anhydrous phase.

### Experimental

A typical hydrothermal synthesis has allowed to isolate the title compound from the reaction mixture of strontium carbonate (SrCO_3_; 0.0738 g), metallic nickel (Ni; 0.0881 g) and 85 wt% phosphoric acid (H_3_PO_4_; 0,10 ml) and water (H_2_O; 10 ml). The hydrothermal reaction was performed in a 23 ml Teflon-lined autoclave under autogeneous pressure at 468 K for two days. The product was filtered off, washed with deionized water and air dried. The resulting product consists of parallelepipedic turquoise crystals besides some gray powder.

### Refinement

All O-bound H atoms were initially located in a difference map and refined with O—H (0.85) distance restraints and a common *U*_iso_ for both H atoms for the water molecule.

### Crystal data

**Table:**

Ni_2_Sr(PO_4_)_2_·2H_2_O	*F*(000) = 416
*M**_r_* = 431.01	*D*_x_ = 3.958 Mg m^−^^3^
Monoclinic, *C*2/*m*	Mo *K*α radiation, λ = 0.71073 Å
Hall symbol: -C 2y	Cell parameters from 454 reflections
*a* = 8.8877 (3) Å	θ = 3.0–27.4°
*b* = 6.0457 (3) Å	µ = 12.99 mm^−^^1^
*c* = 7.3776 (3) Å	*T* = 296 K
β = 114.173 (2)°	Parallelepiped, pale green
*V* = 361.66 (3) Å^3^	0.20 × 0.10 × 0.07 mm
*Z* = 2	

### Data collection

**Table:**

Bruker APEXII diffractometer	454 independent reflections
Radiation source: fine-focus sealed tube	439 reflections with *I* > 2σ(*I*)
graphite	*R*_int_ = 0.028
φ and ω scans	θ_max_ = 27.4°, θ_min_ = 3.0°
Absorption correction: multi-scan (*SADABS*; Bruker, 2005)	*h* = −11→11
*T*_min_ = 0.229, *T*_max_ = 0.403	*k* = −7→7
2376 measured reflections	*l* = −9→9

### Refinement

**Table:**

Refinement on *F*^2^	Primary atom site location: structure-invariant direct methods
Least-squares matrix: full	Secondary atom site location: difference Fourier map
*R*[*F*^2^ > 2σ(*F*^2^)] = 0.019	Hydrogen site location: inferred from neighbouring sites
*wR*(*F*^2^) = 0.052	H atoms treated by a mixture of independent and constrained refinement
*S* = 1.12	*w* = 1/[σ^2^(*F*_o_^2^) + (0.0243*P*)^2^ + 1.4658*P*] where *P* = (*F*_o_^2^ + 2*F*_c_^2^)/3
454 reflections	(Δ/σ)_max_ < 0.001
49 parameters	Δρ_max_ = 0.41 e Å^−^^3^
3 restraints	Δρ_min_ = −0.68 e Å^−^^3^

### Special details

**Table:**

Geometry. All e.s.d.'s (except the e.s.d. in the dihedral angle between two l.s. planes) are estimated using the full covariance matrix. The cell e.s.d.'s are taken into account individually in the estimation of e.s.d.'s in distances, angles and torsion angles; correlations between e.s.d.'s in cell parameters are only used when they are defined by crystal symmetry. An approximate (isotropic) treatment of cell e.s.d.'s is used for estimating e.s.d.'s involving l.s. planes.
Refinement. Refinement of *F*^2^ against ALL reflections. The weighted *R*-factor *wR* and goodness of fit *S* are based on *F*^2^, conventional *R*-factors *R* are based on *F*, with *F* set to zero for negative *F*^2^. The threshold expression of *F*^2^ > σ(*F*^2^) is used only for calculating *R*-factors(gt) *etc*. and is not relevant to the choice of reflections for refinement. *R*-factors based on *F*^2^ are statistically about twice as large as those based on *F*, and *R*- factors based on ALL data will be even larger.

### Fractional atomic coordinates and isotropic or equivalent isotropic displacement parameters (Å^2^)

**Table:**

	*x*	*y*	*z*	*U*_iso_*/*U*_eq_	
Sr1	0.5000	0.5000	0.0000	0.01385 (17)	
Ni1	0.7500	0.7500	0.5000	0.00948 (17)	
P1	0.91308 (11)	0.5000	0.22145 (14)	0.0053 (2)	
O1	1.0211 (2)	0.7094 (3)	0.2739 (3)	0.0110 (4)	
O2	0.7975 (3)	0.5000	0.0027 (4)	0.0143 (6)	
O3	0.7992 (3)	0.5000	0.3363 (4)	0.0084 (5)	
O4	0.6779 (3)	0.5000	0.6298 (4)	0.0128 (6)	
H1	0.577 (4)	0.5000	0.610 (13)	0.15 (4)*	
H2	0.730 (10)	0.5000	0.755 (4)	0.15 (4)*	

### Atomic displacement parameters (Å^2^)

**Table:**

	*U*^11^	*U*^22^	*U*^33^	*U*^12^	*U*^13^	*U*^23^
Sr1	0.0089 (3)	0.0253 (3)	0.0071 (3)	0.000	0.00297 (19)	0.000
Ni1	0.0100 (3)	0.0095 (3)	0.0083 (3)	0.00375 (18)	0.00307 (19)	0.00014 (19)
P1	0.0045 (4)	0.0055 (5)	0.0059 (5)	0.000	0.0020 (3)	0.000
O1	0.0090 (9)	0.0104 (10)	0.0116 (10)	−0.0040 (8)	0.0021 (7)	0.0013 (8)
O2	0.0100 (13)	0.0226 (17)	0.0080 (15)	0.000	0.0014 (11)	0.000
O3	0.0084 (12)	0.0086 (14)	0.0107 (13)	0.000	0.0066 (10)	0.000
O4	0.0076 (13)	0.0211 (17)	0.0107 (14)	0.000	0.0047 (11)	0.000

### Geometric parameters (Å, °)

**Table:**

Sr1—O1^i^	2.626 (2)	Ni1—O1^i^	2.0499 (19)
Sr1—O1^ii^	2.626 (2)	Ni1—O1^vii^	2.0499 (19)
Sr1—O1^iii^	2.626 (2)	Ni1—O3^vi^	2.0895 (18)
Sr1—O1^iv^	2.626 (2)	Ni1—O3	2.0895 (18)
Sr1—O2^v^	2.636 (3)	P1—O2	1.517 (3)
Sr1—O2	2.636 (3)	P1—O1^viii^	1.539 (2)
Sr1—O3	2.797 (3)	P1—O1	1.539 (2)
Sr1—O3^v^	2.797 (3)	P1—O3	1.564 (3)
Ni1—O4^vi^	2.0285 (19)	O4—H1	0.85 (5)
Ni1—O4	2.0285 (19)	O4—H2	0.85 (5)
			
O1^i^—Sr1—O1^ii^	180.0	O1^i^—Ni1—O1^vii^	180.0
O1^i^—Sr1—O1^iii^	96.00 (9)	O4^vi^—Ni1—O3^vi^	85.13 (8)
O1^ii^—Sr1—O1^iii^	84.00 (9)	O4—Ni1—O3^vi^	94.87 (8)
O1^i^—Sr1—O1^iv^	84.00 (9)	O1^i^—Ni1—O3^vi^	90.62 (9)
O1^ii^—Sr1—O1^iv^	96.00 (9)	O1^vii^—Ni1—O3^vi^	89.38 (9)
O1^iii^—Sr1—O1^iv^	180.00 (7)	O4^vi^—Ni1—O3	94.87 (8)
O1^i^—Sr1—O2^v^	76.18 (6)	O4—Ni1—O3	85.13 (8)
O1^ii^—Sr1—O2^v^	103.82 (6)	O1^i^—Ni1—O3	89.38 (10)
O1^iii^—Sr1—O2^v^	103.82 (6)	O1^vii^—Ni1—O3	90.62 (9)
O1^iv^—Sr1—O2^v^	76.18 (6)	O3^vi^—Ni1—O3	180.0
O1^i^—Sr1—O2	103.82 (6)	O2—P1—O1^viii^	110.38 (10)
O1^ii^—Sr1—O2	76.18 (6)	O2—P1—O1	110.38 (10)
O1^iii^—Sr1—O2	76.18 (6)	O1^viii^—P1—O1	110.64 (16)
O1^iv^—Sr1—O2	103.82 (6)	O2—P1—O3	105.67 (16)
O2^v^—Sr1—O2	180.0	O1^viii^—P1—O3	109.83 (10)
O1^i^—Sr1—O3	64.85 (6)	O1—P1—O3	109.83 (10)
O1^ii^—Sr1—O3	115.15 (6)	P1—O1—Ni1^vii^	127.73 (12)
O1^iii^—Sr1—O3	115.15 (6)	P1—O1—Sr1^ix^	121.08 (11)
O1^iv^—Sr1—O3	64.85 (6)	Ni1^vii^—O1—Sr1^ix^	106.29 (8)
O2^v^—Sr1—O3	126.37 (8)	P1—O2—Sr1	104.37 (14)
O2—Sr1—O3	53.63 (8)	P1—O3—Ni1	130.42 (7)
O1^i^—Sr1—O3^v^	115.15 (6)	P1—O3—Ni1^x^	130.42 (7)
O1^ii^—Sr1—O3^v^	64.85 (6)	Ni1—O3—Ni1^x^	92.66 (11)
O1^iii^—Sr1—O3^v^	64.85 (6)	P1—O3—Sr1	96.33 (13)
O1^iv^—Sr1—O3^v^	115.15 (6)	Ni1—O3—Sr1	99.48 (8)
O2^v^—Sr1—O3^v^	53.63 (8)	Ni1^x^—O3—Sr1	99.48 (8)
O2—Sr1—O3^v^	126.37 (8)	Ni1^x^—O4—Ni1	96.34 (12)
O3—Sr1—O3^v^	180.00 (9)	Ni1^x^—O4—H1	116 (3)
O4^vi^—Ni1—O4	180.0	Ni1—O4—H1	116 (3)
O4^vi^—Ni1—O1^i^	85.80 (10)	Ni1^x^—O4—H2	112 (4)
O4—Ni1—O1^i^	94.20 (10)	Ni1—O4—H2	112 (4)
O4^vi^—Ni1—O1^vii^	94.20 (10)	H1—O4—H2	105 (8)
O4—Ni1—O1^vii^	85.80 (10)		

Symmetry codes: (i) *x*−1/2, −*y*+3/2, *z*; (ii) −*x*+3/2, *y*−1/2, −*z*; (iii) −*x*+3/2, −*y*+3/2, −*z*; (iv) *x*−1/2, *y*−1/2, *z*; (v) −*x*+1, −*y*+1, −*z*; (vi) −*x*+3/2, −*y*+3/2, −*z*+1; (vii) −*x*+2, *y*, −*z*+1; (viii) *x*, −*y*+1, *z*; (ix) *x*+1/2, *y*+1/2, *z*; (x) −*x*+3/2, *y*−1/2, −*z*+1.

### Hydrogen-bond geometry (Å, °)

**Table:**

*D*—H···*A*	*D*—H	H···*A*	*D*···*A*	*D*—H···*A*
O4—H1···O4^xi^	0.85 (6)	2.23 (7)	2.951 (4)	143 (7)
O4—H1···O1^vi^	0.85 (6)	2.28 (4)	2.784 (3)	118 (4)
O4—H1···O1^x^	0.85 (6)	2.28 (4)	2.784 (3)	118 (4)
O4—H2···O2^xii^	0.85 (3)	1.67 (3)	2.511 (4)	169 (9)

Symmetry codes: (xi) −*x*+1, −*y*+1, −*z*+1; (vi) −*x*+3/2, −*y*+3/2, −*z*+1; (x) −*x*+3/2, *y*−1/2, −*z*+1; (xii) *x*, *y*, *z*+1.
